# Current trend of accreditation within medical education

**DOI:** 10.3352/jeehp.2020.17.30

**Published:** 2020-10-21

**Authors:** Ducksun Ahn

**Affiliations:** Professor Emeritus, Korea University College of Medicine, Seoul, Korea; Hallym University, Korea

**Keywords:** Accreditation, Goals, Medical education, Quality improvement, Training support

## Abstract

Currently, accreditation in medical education is a priority for many countries worldwide. The World Federation for Medical Education’s (WFME) launch of its 1st trilogy of standards in 2003 was a seminal event promoting accreditation in basic medical education (BME) globally. In parallel, the WFME also actively spearheaded a project to recognize accrediting agencies within individual countries. The introduction of competency-based medical education (CBME), with the 2 key concepts of entrusted professional activity and milestones, has enabled researchers to identify the relationships between patient outcomes and medical education. The recent data-driven approach to CBME has been used for ongoing quality improvement of trainees and training programs. The accreditation goal has shifted from the single purpose of quality assurance to balancing quality assurance and quality improvement. Although there are many types of postgraduate medical education (PGME), it may be possible to accredit resident programs on a global scale by adopting the concept of CBME. It will also be possible to achieve accreditation alignment for BME and PGME, which center on competency. This approach may also make it possible to measure accreditation outcomes against patient outcomes. Therefore, evidence of the advantages of costly and labor-consuming accreditation processes will be available soon, and quality improvement will be the driving force of the accreditation process.


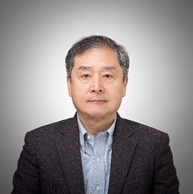


## Introduction

### Background/rationale

Accreditation, as a means to ensure the quality of higher education, is a global concern. The World Health Organization requires countries to establish accreditation bodies for quality management of education to train high-quality medical personnel [1]. Accordingly, the World Federation for Medical Education (WFME) is recognizing the accreditation bodies for basic medical education in each country as its most critical project. The WFME, along with the Educational Commission for Foreign Medical Graduates (ECFMG) in the United States, is also actively working on a global medical school directory. This work aims to build an international information network for providers of basic medical education and to improve providers’ transparency [2]. Medical education can be divided into basic medical education, postgraduate medical education for interns and residents, and lifelong professional education. The WFME has led the accreditation standards of medical schools around the world; therefore, it is necessary to carefully examine the WFME’s work.

### Objectives

This study aimed to describe global changes related to the accreditation of basic medical education, postgraduate education, and lifelong professional education. Some documents were cited based on issues regarding accreditation. Furthermore, global changes in accreditation were described.

## Activities of the World Federation for Medical Education

The WFME is an independent public organization established in 1972 by the World Health Organization and the World Medical Association [3]. Since Korea dispatched many participants to the WFME World Conference in 2003, the WFME has become well known in Korea as a global medical education organization. In 2003, the global standards for basic medical education, postgraduate medical education, and continuous professional development were published as the “WFME Trilogy.”

In recent years, full membership on the WFME’s Executive Committee has been provided to 2 networks: the International Federation for Medical Students Association (IFMSA) and Junior Doctors Network. IFMSA, as an organization of students , requested the WFME and the ECFMG of the United States to publish the accreditation status of all medical schools listed in the World Directory of Medical Schools. This list of medical schools throughout the world is expected to describe each medical school’s state of accreditation. The former WFME chairman Karle [4] has warned about the appearance of commercial medical schools. Additionally, more applicants are applying to medical schools throughout the world. In response to this phenomenon, internationally shared medical education standards are needed. It is therefore time to disclose transparent educational information on all medical schools throughout the world.

Education providers have a social responsibility to disclose the status of their accreditation. Twenty-three accreditation bodies, including the Korean Institute of Medical Education and Evaluation (KIMEE), have obtained official recognition from the WFME as of September 2020. Accreditation bodies in many countries are preparing for official recognition by the WFME.

## Accreditation criteria of the World Federation of Medical Education

The WFME published accreditation criteria for the 3 stages of medical education in 2003: basic medical education, postgraduate education, and continuing professional development (https://wfme.org/standards/). Subsequently, in 2012 and 2015, a revised edition for basic medical education was published, and detailed descriptions of the standards were strengthened. Currently, the WFME’s Standards Development Committee is transitioning to a more user-friendly and more straightforward standard for non-English-speaking countries. It is expected that the latest updated basic medical education standards will be published soon.

In addition to the user-friendly point of view of the accreditation criteria, a user-friendly perspective on accreditation methods and procedures is also emphasized. According to Barzansky [5], a representative of the Liaison Committee on Medical Education (LCME), an American medical school accreditation body, the LCME is also seeking to establish a user-friendly accreditation system for medical schools. The LCME has developed a platform to easily input a large amount of data relevant for medical schools’ accreditation.

## Aging society and changes in accreditation criteria

Health care or medical education is determined by various factors such as the economy, politics, society, and culture. At the end of the 1st decade of the 21st century, population aging was identified as a common international issue affecting health care. The United Nations is raising awareness about the international trend towards aging and demanding action from each country [6]. Korea has already become an aging society due to the rapid increase in the elderly population. The integration of medical care and welfare for the aging population is emerging as an essential social and political issue. The Korean government is trying to benchmark the Japanese integrated care model, which is called “Comcare (integrated care),” and to introduce medical treatment to cope with the aging society in a similar manner [7]. Japanese-style Comcare is rooted in the principle of integrated care, as social welfare and medical care, including housing, have been integrated into a single service in Europe, which already became an older society a generation ago [8]. In Europe, 25% of the population is currently aged 60 and older. Since all developed countries have entered the era of aging societies, integrated care is now emerging as a basic, inevitable form of medical care in societies with aging populations. For integrated care to be implemented, integrated coordination and management between multiple stakeholders of care are necessary. Some countries already reflect this through interprofessional education in medical school accreditation standards. Zorek and Raehl [9] published comparative analyses of interprofessional education in other fields such as nursing, dentistry, pharmacy, and medical schools, and showed that an interprofessional approach has already become common in health care education.

Korea, which is now becoming an aging society, is attempting to introduce integrated care within the framework of community care. However, no medical school has yet attempted to implement a full-fledged educational program to prepare for community care. Interprofessional education has not been actively implemented in Korea, because Korea has only recently entered the era of an aging society in earnest. It is expected that governmental integrated care initiatives will become more fully operational within only a year or so. An appropriate medical school curriculum and accreditation standards have not yet been established to address integrated care and interprofessional education. As in other developed countries, integrated care for the elderly will likely be adopted as a criterion for basic medical education accreditation.

## Changes in emergency medicine

Another common phenomenon resulting from the increasing number of elderly patients in many countries that have become an aging society is that the emergency room is gradually becoming an elderly care center. Some countries also have separate accreditation for elderly care in emergency medicine. Hwang et al. [10] described the phenomenon of caring for the elderly in the emergency room. They reported that when physicians treated elderly patients following the same workflow as in conventional emergency care, the probability that they would return to the emergency room within a month was more than 25%. Thus, emergency room treatment for an elderly patient is regarded as unnecessary medical care and a waste of medical resources if he or she is not is provided with integrated care linked to the community at the time of discharge.

Older people often have multiple diseases simultaneously, and their treatment and management can therefore be time-consuming. As a result, physicians increasingly refer older patients to emergency rooms due to difficulties involving overtime or weekend care. The American Emergency Medical Association mandates multidisciplinary health and medical professional training for the elderly as part of a separate accreditation standard for elder care in the emergency room [11]. To coordinate the integrated care for elderly patients, the emergency room must also have a senior evaluation team of representatives from 2 to 4 professions. In medical education, understanding the necessary coping skills and integrated care for the elderly is considered essential. This competency can be added at any time as part of the basic medical education standard.

## Strengthening of universal health coverage and the emergence of health systems science

Strengthening of universal health coverage is a consistent goal of the World Health Organization. However, increasing medical expenses for elderly patients, new and advanced technological advances, and pharmaceutical expenses have led to rising medical expenses. Many developed countries are already paying more than 10% of their gross domestic product for health care. Skochelak et al. [12] introduced health systems science (HSS) as the 3rd pillar of medical school education, and it is expected that accreditation standards and related curricula will be established regarding HSS in the future. HSS was developed to supplement the limitations of providing cost-effective medical services while achieving the goals of high-quality medical care and health promotion. Therefore, it has been identified as a social necessity that future medical personnel should have the ability to make rational decisions from the standpoint of HSS, as well as the clinical and basic capabilities for traditional patient care.

## Development of competency-based medical education

The concept of entrustable professional activity (EPA) advocated by ten Cate [13] and the concept of milestone stages by Dreyfus et al. [14] has been discussed as a basic principle of competency-based medical education. Frank et al. [15] explained the theory and practice of competency-based education methodology in medical education through international cooperation. They also suggested that competency-based medical education should become the educational methodology of the 21st century. The introduction of the 2 core concepts of EPA and key milestone stages in competency-based medical education has opened the door for research exploring correlations between undergraduate education and residency programs and trainees’ outcomes after acquiring specialty board certification. The relationship between current medical education accreditation and clinical outcomes has opened the possibility for further directions of research. New methodologies in medical education and a wide variety of detailed learning evaluation methods are revolutionizing the framework of the accreditation. At the WFME World Congress held in Korea in April 2019, the United States and Canada already demonstrated progress towards accreditation using the concepts of EPA and key stages [16].

## Accreditation and development of competency-based medical education

Until recently, the high cost of accreditation and the labor-intensive nature of the accreditation or evaluation of medical schools have been criticized by medical schools, government, and politicians who pay for these costs. Davis and Ringsted [17] argued that accreditation of educational methodologies and curricula itself has little effect on educational outcomes. Furthermore, they pointed out that the results of accreditation focus on inputs and processes, whereas the outcomes of education are unclear. The link between the results of accreditation of postgraduate education and outcomes is likewise unclear. Contrary to the above-mentioned study that expressed a negative perspective on the effectiveness of accreditation, van Zanten et al. [18], in a study conducted in the Philippines and Mexico, showed that medical students from accredited medical schools were more likely to pass the US medical licensing examination than those from non-accredited schools. This finding supports the proposal that accreditation has positive effects.

Since the 1970s, the World Health Organization has insisted on the necessity of competency-based medical education [1,19], but methods to put it into practice were insufficient. However, EPA, the core concept of competency-based medical education, and the development of the milestone stage concept led to a new era of competency-based medical education. The effects of accreditation of basic medical education can be analyzed by measuring graduates’ clinical performance. Another aspect of competency-based medical education is that continuous quality management in medical education has become more specific and systematic. Shojania et al. [20] have presented various methodologies for improving quality in medicine. This combination of research on quality improvement and medical education based on competency enabled the development of concrete methodologies for continuous quality improvement in medical education. The link between basic medical education and postgraduate education has been considered as a task for the future.

## Accreditation and development of continuous quality improvement

Now, we must go beyond the quality assurance of education, which was the original purpose of accreditation. It is time for each medical school to promote the quality of the entire medical education cycle by issuing annual reports on basic and postgraduate medical education. Barzansky et al. [21] argued for the importance of accreditation with periodic and comprehensive evaluations. Self-evaluation reports are submitted annually. Based on these annual reports, ongoing efforts should be made to strengthen weak areas of competencies. The new era of pursuing continuous quality improvement in medical education has been made possible by this approach. Since 2012, the KIMEE has required each medical school in Korea to submit an interim report every 2 years after certification. The interim report is also reviewed separately. To continue improving the quality of medical education, priorities should include data sharing and the formation of a data network for all medical education providers at the national level. Based on these data, the KIMEE can conduct research on accreditation. Furthermore, quality assurance of medical education and quality improvement can be pursued based on the collected data.

## Changes in the function of accreditation: balancing quality improvement and quality assurance

Quality improvement has emerged as a major issue in residents’ education as basic medical education requires periodic submission of self-evaluation reports for continuous quality improvement. The Accreditation Council for Graduate Medical Education (ACGME) in the United States can analyze residents’ development and the characteristics of residency programs, as the evaluation of residents’ competency has become more sophisticated. It has also become possible obtain a snapshot of the landscape of any specific type of residency at the national level [22]. The importance of a 360-degree multi-faceted evaluation by all possible stakeholders, including doctors, nurses, staff, patients, and students, is being emphasized as part of the evaluation of physicians. However, due to the cultural characteristics of Korea, it has been difficult to introduce initiatives of this type. The competency framework for residents has already been established in some developed countries, and it is possible to measure competency outcomes as a way of assessing resident training programs. The World Medical Association has recognized the importance of continuous quality improvement and announced its policy on guidelines for continuous quality improvement in health care [23].

## Efforts for continuous quality improvement after obtaining a license

In September 2019, a symposium of the International Association of Medical Regulatory Authorities was held in the United States [24]. Representatives from the Medical Council of Canada, the Canadian College of Physicians and Surgeons of Canada, and the medical regulatory authorities in Australia, New Zealand, and the United Kingdom presented common topics: namely, assurance of the quality of medical education for the acquisition of licenses and maintenance of physicians’ competencies after they are licensed. The ACGME in the United States has an accredited residency program with 6 core competencies that apply the concept of key stages of performance-based evaluation [25]. Regarding residency programs’ outcomes, various aspects of practice characteristics have been documented according to the programs completed by residents. For instance, Asch et al. [26] presented links between complications and patient outcomes reported by gynecologists.

Furthermore, Sirovich et al. [27] showed a relationship between conservative medical management and internal medicine residency programs. Chen et al. [28] also reported a correlation between resident education and medical expenses. Smirnova et al. [29], in the Netherlands, published a research report on the correlation between obstetrics and gynecology education and complications. These studies have revealed correlations between aspects of residency programs and medical outcomes after residents become specialists.

## Conclusion

With the ongoing efforts of the WFME, the project of accreditation of institutes for basic medical education in each country is moving forward. After the WFME World Congress 2019, the WFME is considering accreditation for trainees in transitional periods, such as interns or clinical practice students, to be licensed. As the 1st step for such accreditation programs, it is necessary to grasp the international status and develop general competency standards, but such initiatives are still in the discussion stage. Because of the variety of systems, histories, and forms of education for resident education from country to country, internationally standardized accreditation is still premature. However, it may soon be possible to achieve international accreditation based on the concept of competency. Currently, the WFME is considering a methodology for international accreditation of residents’ education.

Regarding the accreditation of continuing professional development, the US Accreditation Council for Continuing Medical Education (ACCME) has already begun to do this job [30]. The Korea Medical Association’s Council on Continuing Medical Education is considering applying for official international accreditation by the ACCME as a national accreditation agency.

International efforts for accreditation in basic medical education, postgraduate education, and continuing professional development, as well as new medical education initiatives, are strengthening interconnections throughout the cycle of artificially segmented medical education. These developments also provide clues regarding the scientific interpretation of links between medical education and the outcomes of medical practice. A new chapter of medical research is opening to explore correlations between patients’ clinical outcomes and aspects of medical education. Based on this evidence, the accreditation process aims to achieve 2 balanced purposes: quality assurance and quality improvement.

## Supplementary materials

Supplement 1. The audio recording of the abstract.
